# Public-private relationship in surgical hospitalizations through the Unified Health System

**DOI:** 10.1590/1518-8345.4901.3467

**Published:** 2021-08-30

**Authors:** Liane Alves de Sá, Eduardo Rocha Covre, Willian Augusto de Melo, Rogério Miranda Gomes, Maria Fernanda do Prado Tostes

**Affiliations:** 1 Universidade Estadual do Paraná, Colegiado de Enfermagem, Paranavaí, PR, Brazil.; 2 Scholarship holder at the Fundação Araucária, Universidade Estadual do Paraná, Brazil.; 3 Universidade Federal do Paraná, Departamento de Saúde Coletiva, Curitiba, PR, Brazil.

**Keywords:** Operative Surgical Procedures, Unified Health System, Government Financing, Public Sector, Private Sector, Public Health Nursing, Procedimentos Cirúrgicos Operatórios, Sistema Único de Saúde, Financiamento Governamental, Setor Público; Setor Privado, Enfermagem em Saúde Pública, Procedimientos Quirúrgicos Operativos, Sistema Único de Salud, Financiación Gubernamental, Sector Público; Sector Privado, Enfermería en Salud Pública

## Abstract

**Objective:**

to characterize surgical hospitalizations, length of stay, cost and mortality, according to the legal nature (public and private) of the hospital institution linked to the Unified Health System (*Sistema Único de Saúde*, SUS). **Method**: a descriptive study, of the survey type, with retrospective data collection (2008 to 2017) and a quantitative approach. The dependent variables surgical hospitalizations in Brazil, costs, length of stay and mortality and the independent variables regime/legal nature (public and private) were obtained from the Informatics Department of the Unified Health System. The *Mann-Whitney* test was used for analysis.

**Results:**

the average number of hospitalizations through the Unified Health System was 4,214,083 hospitalizations/year, 53.5% occurred in private hired hospitals and 46.5% in public hospitals (p=0.001). The financial transfer was greater for the private sector (60.6%) against 39.4% for the public (p=0.001). The average stay was 4.5 days in the public hospital and 3.1 days in its private counterpart (p<0.001). Mortality was higher in the public (1.8%) than in the private hospital (1.4%) (p<0.001).

**Conclusion:**

there was predominance of surgical hospitalizations through the Unified Health System in private hospitals with greater financial transfer to this sector, to the detriment of the public. The diverse evidence produced contributes to the debate and actions to avoid budgetary asphyxiation in the public sector in favor of the private sector.

## Abstract (pt)

**Objetivo:**

caracterizar as internações cirúrgicas, permanência, custo e mortalidade, conforme a natureza jurídica (público e privado) da instituição hospitalar ligada ao Sistema Único de Saúde. **Método**: estudo descritivo, tipo levantamento, com coleta de dados retrospectiva (2008 a 2017) e abordagem quantitativa. As variáveis dependentes internações cirúrgicas ocorridas no Brasil, custos, permanência e mortalidade e as variáveis independentes regime/natureza jurídica (público e privado) foram obtidas do Departamento de Informática do Sistema Único de Saúde. O teste de Mann-Whitney foi empregado para análise.

**Resultados:**

a média das internações pelo Sistema Único de Saúde foi de 4.214.083 internações/ ano, 53,5% ocorridas nos hospitais privados contratados e 46,5% em hospitais públicos (p=0,001). O repasse financeiro foi maior para o setor privado (60,6%) contra 39,4% para os públicos (p=0,001). A média de permanência foi de 4,5 dias no público e 3,1 dias no privado (p<0,001). A mortalidade foi maior no público (1,8%) do que no privado (1,4%) (p<0,001).

**Conclusão:**

houve predomínio das internações cirúrgicas pelo Sistema Único de Saúde nos hospitais privados com repasse financeiro maior para este setor em detrimento do público. As evidências produzidas contribuem para o debate e ações para evitar o asfixiamento orçamentário do setor público em privilégio do privado.

## Abstract (es)

**Objetivo:**

caracterizar los ingresos quirúrgicos, la estancia, el costo y la mortalidad, según la naturaleza jurídica (público y privado) del hospital vinculado al Sistema Único de Salud.

**Método:**

se trata de estudio descriptivo, tipo encuesta, con recolección de datos retrospectiva (2008 a 2017) y enfoque cuantitativo. Las variables dependientes ingresos quirúrgicos en Brasil, costos, estancia y mortalidad y las variables independientes régimen/naturaleza jurídica (público y privado) se obtuvieron del Departamento de Informática del Sistema Único de Salud. Para el análisis se utilizó la prueba de MannWhitney.

**Resultados:**

el promedio de hospitalizaciones a través del Sistema Único de Salud fue de 4.214.083 hospitalizaciones/ año, 53,5% ocurrieron en hospitales privados contratados y 46,5% en hospitales públicos (p=0,001). La transferencia financiera fue mayor para el sector privado (60,6%) y del 39,4% para el público (p=0,001). La estancia media fue de 4,5 días en el público y 3,1 días en el privado (p<0,001). La mortalidad fue mayor en los hospitales públicos (1,8%) que en los privados (1,4%) (p<0,001).

**Conclusión:**

hubo un predominio de los ingresos quirúrgicos a través del Sistema Único de Salud en los hospitales privados con mayor transferencia económica a este sector en detrimento de la población. La evidencia producida contribuye al debate y las acciones para evitar la asfixia presupuestaria del sector público que privilegia al sector privado.

## Introduction

Affordable and safe surgical assistance is essential to reduce morbidity and mortality and disabilities resulting from surgical conditions. In addition to that, it improves the well-being of the population, economic productivity, and the capacity and freedom of the individuals, contributing to the long-term development of the countries^(1)^.

Despite this importance, access to surgery is not fully guaranteed to the population, especially in peripheral countries or without universal health systems, contributing to the occurrence of complication in cases that could be resolved with less complex surgeries^(2)^.

In the global context, it is estimated that approximately five billion people do not have access to essential, inexpensive and safe surgical and anesthetic care performed in a timely manner^(1)^, which makes it difficult to strengthen health systems and Universal Access to Health^(3)^.

According to the World Health Organization, Brazil is characterized as a middle-income country^(4)^ and the context about guaranteeing access is no different from the aforementioned world scenario. Several studies show that, in the country, the surgical volume is lower than that recommended by the Lancet Commission on Global Surgery goal, which should be 5,000 *per* 100,000 inhabitants/year by 2030^(5-6)^. From 2008 to 2016, the mean surgical volume was 2,020 surgeries *per* 100,000 inhabitants/year performed by the Unified Health System (*Sistema Único de Saúde,* SUS)^(6)^.

In Brazil, it is worth noting that, in order to guarantee the population’s access to the health services, including surgical procedures, in addition to the use of federal, state and municipal public services, SUS also hires private services, which include profit and non-profit institutions and philanthropic institutions in a complementary manner, as provided for in the 1988 Constitution^(7)^.

In this perspective, the scarcity of studies conducted on how surgical hospitalizations are distributed by SUS is highlighted, focusing on the legal nature of the hospital units, including all surgical specialties and of national geographic scope. In fact, an existing study with this focus was limited to addressing the public-private arrangement in only one surgical specialty^(8)^. Therefore, this study is a precursor in the production of this knowledge and can contribute to reducing the knowledge gaps and to the scientific advancement of this theme in Health and Nursing.

In view of the above, the following question was asked: How are surgical hospitalizations distributed nationwide by SUS and other related variables in relation to the legal nature of the hospitals? In order to answer the research question, this study aimed to characterize surgical hospitalizations, length of stay, costs and mortality, according to the legal nature (public and private) of the hospital institution linked to the Unified Health System.

## Method

### Type of study

This is a descriptive, survey-type study with a quantitative approach.

### Data collection place

The secondary source information was obtained through the database of the SUS Informatics Department (DATASUS)^(9)^. And, in the System, the geographic scope established was Brazil.

### Variables

The dependent variables of the study were surgical hospitalizations, average length of stay, mean value of the hospitalization, value of the hospital service, and mortality rate.

The independent variables considered were the regime and legal nature of the hospital unit hired by SUS: public and private.

### Data collection period

In the DATASUS, retrospective data collection took place in July 2018. To obtain the study variables, the period from 2008 to 2017 was considered. The initial period considered was 2008 because, in the DATASUS system, the consolidated data since 2008 provide more specific information, such as services and procedures performed, as well as groups and subgroups of these procedures^(9)^. And the complete annual data were available in the System until 2017; as a result, this time limit was established.

### Data collection

In the DATASUS system, in the Health Information option (TABNET), the Health Care option was selected, followed by the Hospital Production option (SIH/SUS) and the Consolidated Data option, by place of hospitalization, starting in 2008. At this moment, the geographical coverage selected was Brazil by Region and Federation Unit.

Subsequently, in the row option, the Year of Processing was selected, in the column the Regime option for data collection referring to the period between 2008 and 2014, and Legal Nature, for data collection referring to the period from 2015 to 2017. It is noted that the use of the two options Regime and Legal Nature in the data collection process was necessary because, in the System, the Regime and Nature classifications are available for processing until 2014. Since 2015, the classification of Legal Nature and Legal Sphere is used.

Then, in the content option, each study dependent variable was selected separately. And in the Available Selections section, the option Group Procedures was selected, and the option Surgical Procedures was demarcated.

### Data treatment and analysis

After collection in the DATASUS, the results were tabulated in data spreadsheets in the *Microsoft Excel* software, version 2007. Precisely on the dependent variables related to the costs, the values were converted to US dollars, considering the equivalence on July 20^th^, 2018 (period of data collection) = 3.7787 reais.

For the analysis, the Mann-Whitney statistical test was used using the Statistica program, version 10. In the analyses, a 95% confidence interval and 5% significance level (p<0.05) were established. The data were presented in a table and boxplot graphs.

### Ethical aspects

The study was approved by the Ethics Committee, CAAE number 14956719.6.0000.9247 and approval opinion number 3,387,441/2018.

## Results

In the DATASUS, in ten years, 42,140,832 surgical hospitalizations through the SUS were registered (annual average of 4,214,083 hospitalizations). Of this number, 53.5% (22,543,816) occurred in private hospitals hired by the SUS, while in public hospitals they were 46.5% (19,594,158). And in 2,858 surgical hospitalizations, the regime/legal nature was not specified.

Regarding hospital stay, the average was 3.8 days. In the public sector it was higher (4.5 days) than in the private sphere (3.1 days).

The cost of the hospital services was 11,823,847,361.41 dollars; private hospitals received 7,165,240,353.88 dollars while public hospitals received 4,658,607,007.53 dollars, representing 60.6% and 39.4% of the costs, respectively.

The average value of hospitalization was 368.57 dollars; this amount was more substantial in the private sphere (415.63 dollars) than in the public sphere (312.86 dollars).

In relation to mortality, the general rate established was 1.6%; this index was higher (1.8%) in public hospitals than in private hospitals (1.4%).

**Table 1:** Distribution of the surgical hospitalizations through the Unified Health System (2008 to 2017), hospital stay, costs and mortality, differentiated between public and private. Brazil, 2018*

Variables	Values	Average	Standard Deviation	Variation Coefficient	**Mann-Whitney test (p-value)^†^**
**Hospitalizations** Public Sector	19,594,158	1.95	2.0	11.22375	0.001
Private Sector	22,543,816	2.25	1	2.40148	
**Average Stay** Public Sector	4.5	4.5	0	2.0512	<0.001
Private Sector	3.1	3.1	0	1.52066	
**Hospital Service Value**^‡^ Public Sector	4,658,607,007.53	4.65	3.4	19.75933	0.001
Private Sector	7,165,240,353.88	7.16	5.5	20.33038	
**Average Hospitalization Value**^‡^ Public Sector	312.86	3.12	118	9.98838	0.005
Private Sector	415.63	4.15	295	18.78842	
**Mortality Rate** Public Sector	1.8	1.81	0	2.11316	<0.001
Private Sector	1.4	1.43	0	6.19564	

^*^Source: Ministry of Health, Informatics Department of the Unified Health System, 2018; ^†^p-value<0.05 considered statistically significant; ^‡^US dollar equivalence = R$ 3.7787 on 07/20/2018

Regarding the differentiation between public and private, there was a statistically significant difference (p<0.05) in all the variables analyzed, as shown in Table 1.

**Figure 1:**
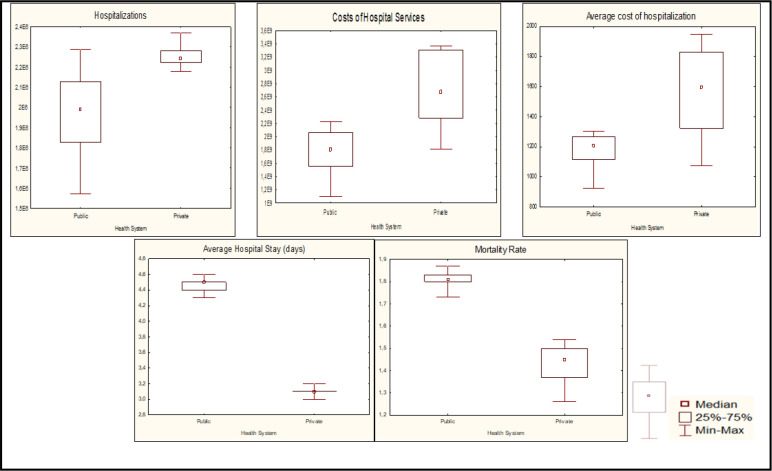
Boxplot of the surgical hospitalizations through the Unified Health System (2008 to 2017), average hospital stay, mortality rate, average hospitalization value and value of the hospital services differentiated between public and private. Brazil, 2018

In relation to Figure 1 (boxplot), the analysis of the distribution of surgical hospitalizations presented a higher median in the private sector (2.24) than in the public sector (1.99). The median on the value of the hospital services variable was more expressive in the private sector (2.6) than in the public sector (1.8). Regarding the average value of surgical hospitalizations, the medians were 1.6 in the private and 1.2 in the public.

However, with regard to the average length of stay and the mortality rate, there are more expressive values in the public when compared to the private sector. The values include the following: median of 4.5 for the public sector and of 3.1 for the private sector, referring to the average length of stay, while the median on the mortality rate is 1.8 in the public and 1.4 in the private sectors, respectively. private.

## Discussion

In this study, it can be verified that, in the tenyear period, the hospitalizations for surgical procedures by SUS occurred more in private hospitals than in their public counterparts, with a statistically significant difference between both. Additionally, the average value of hospitalization and the total value of the hospital services in the period were higher in the private sphere, showing the remarkable transfer of public resources to this sector. In fact, in hospitalizations for surgical procedures, it can be asserted that the public services are complementary to the private ones.

Similarly to the aforementioned results, in a study conducted to discuss the public-private relationship in high-complexity cardiovascular care in SUS, the authors evidenced that 73% of the services were provided by private entities hired by SUS^(8)^.

In Brazil, the number of health institutions rose from 21,532 in 1981 to 129,544 in 2017, a growth led by the expansion of Clinics and Support Services for Diagnosis and Therapy (*Serviços de Apoio ao Diagnóstico e Terapia*, SADTs) and Basic Health Units (BHUs). However, while the BHUs are predominantly public (99.2% in 2017), in the SADT units, the private presence stands out (86.8% in 2017). During this period, there was a progressive decline in public participation, since in 1981 it exceeded the percentage of 50%^(10)^.

Regarding the hospital environment, the number of hospitals rose from 5,660 in 1981 to 8,139 in 2019^(11)^. The Brazilian historical trend has been of an approximately equivalent distribution among public hospitals (approximately one third of hospitals), profitable private ones (also around one third) and philanthropic private institutions (idem)^(12)^. The total number of hospital beds in the country (including those hired and not hired by SUS) decreased from 460,656 in 2008 to 437,565 beds in 2018, with this reduction occurring fundamentally among private beds hired by SUS^(13)^.

This dynamics, of reducing the number of beds and increasing the number of hospitals, demonstrates a reduction in the mean size of the Brazilian hospitals in the last decades, with public hospitals having a mean number of beds lower than private ones^(12)^. Public beds increased in the period, but remain as a minority, representing only 35.8% of the total number of beds in the country in 2017^(10)^. With that, currently, more than half of the hospitalizations by SUS remain in private hospitals, whether profitable and private or the so-called philanthropic institutions.

It is also worth mentioning the progressive decline in the number of beds *per* thousand inhabitants, from 4.1 in 1976, to 3.2 in 1995, and reaching 2.3 in 2019^(11-12)^. Considering only the general beds available for the SUS, there were 0.91 beds *per* thousand inhabitants in 2019. The rate of hospitalizations in the country has also declined since the implementation of SUS, related to changes in the care model and to policies to contain hospital expenses^(12)^.

In the Brazilian health system, hospital care is predominantly provided by private services, simultaneously serving users of the SUS and private insurance, implying funding provisions and complex assistance arrangements that hinder health integration and system regulation^(14)^.

In recent years, the transfer of the provision of public services to the private sector through various mechanisms such as outsourcing, public-private partnerships and Social Organizations (SOs), among others, has played an increasing role. Several authors point out that these mechanisms can lead to the distortion of the assumptions that define the health needs, favoring market interests^(15-16)^.

In fact, the large number of public resources invested in private health contributes to the commercialization of health, making it interesting to investors, through tax exemptions, among other financial mechanisms^(16-17)^. This logic directs social protection institutions and policies to serve the interests of accumulation, enabling capital to commercialize virtually all dimensions of social life, including health.

Regarding the values of the surgical hospitalizations, the results show that the procedures in the private network cost more than in the public one. In a study conducted in order to evaluate the public-private arrangement in the scope of cardiovascular care between 2008 and 2015, the authors also evidenced that the hospitalizations in private institutions funded by the SUS exceeded those that occurred in public hospitals and were more expensive for the system^(18)^.

In addition to that, the private sector selects the most profitable services and procedures according to the remuneration in the SUS table^(15)^. Consequently, this dynamics of public-private division in procedures, services and remuneration values guided by market interests configure an important determination of the limits to the universality of access to health^(16)^.

The definition of the SUS Table as a reference and not as a limit also opens room for higher prices to be established by private providers, as it is common in contracts with municipal and state entities.

In a qualitative study conducted with municipal managers to analyze the relationship between public managers and private providers hired by SUS, the authors verified that the relationship is often tense, compounded by advantages, privileges and interests, and characterized by unequal power relationships, which are made possible by the *modus operandi* of both actors involved (managers and providers). In this relationship, the predominant logic is the search for profitability, in addition to political physiologism; the adaptation of managers and the perception of the absence of alternatives also contribute to the maintenance of this dynamics^(15)^.

Regarding the average hospital stay and the mortality rate, the results evidenced that the highest values were in the public sector. Another study, which investigated whether adjusted hospital mortality differs according to the source of payment for the hospitalizations, legal nature and funding arrangement of the hospitals, analyzed 852,864 hospitalizations in adults, in 789 hospitals between 2008 and 2010, pointing out that the number of deaths of patients funded by the SUS was higher when compared to patients who have health insurance or paid privately. The implications of the funding type and the legal nature of the service on adjusted mortality were not significant. However, even if these aspects were not associated with differentials in the risk of death, within the same hospitals, the existence of different physical structures was noticed and, possibly, of resources, for SUS and non-SUS patients, indicating inequalities in the care process^(14)^.

In general, it is noticed that the selection of more profitable procedures leads to the tendency of concentration of private providers in the most profitable spheres, seeking better paid interventions and with shorter hospital stays, enabling greater productivity with the installed capacity. Often, the most chronic conditions, with longer stays, higher costs and higher mortality rates have predominantly depended on the public hospitals^(12)^. This trend is even present in other public systems with a strong private presence, such as the French social insurance^(19)^.

Although not analyzed in this study, possible differences in performance between public and private health services were object of analysis. In Brazil, even though there are few studies on the theme, and rarely conclusive, international studies in general point to the superiority of universal public systems with strong participation and state regulation and based on Primary Health Care as a gateway and care coordinator^(3)^.

In this sense, several authors point out that this excessive hiring of private services is in opposition to the SUS principles and guidelines, since it impairs care integration, longitudinality and user access^(17,20)^. Far from being a Brazilian dilemma, the obstacles to comprehensive care resulting from the public-private mix cover several countries, especially in Latin America^(21)^.

Although SUS constitutes an important universal system, the contradictory and complexly intertwined coexistence with the private sector has hindered the realization of the universal right to health^(3)^. Among the problems arising from privatization, tax deductions and exemptions for private institutions (profitable and philanthropic), high administrative costs with outsourcing and corruption stand out. In addition to that, the financialization and internationalization of the health sector has advanced significantly in recent decades, through processes of acquisition-merger of companies, directed by financial funds, with important implications for the raising of public funds, capital appreciation and institution of monopoly pricing. This advance in privatization and the fact that these actors have representatives in government decision-making centers has been the subject of virtually no reflection by social participation entities and health councils^(16)^.

To this worrying situation we must add the fact that, since its creation, SUS has presented itself with restricted public funding, relative to Gross Domestic Product (GDP), when compared to other countries with universal health systems^(22)^. As a result, several studies have highlighted some key elements to prevent the dismantling of the public system through privatization and underfunding^(20,22-24)^.

On the one hand, the need to overcome the chronic underfunding of the SUS involves, in addition to the definition of new sources of financial resources, the prohibition of transferring the public fund to private insurance and companies, the end of the tax waiver for health plan operators, as well as greater State regulation on supplementary health^(20)^.

In addition to that, in order to guarantee comprehensive care, it is fundamental to expand the health reform to the hospital services, through their integration into the network as a territorial reference and support for Primary Care and for medium-complexity and urgency services. In this, the constitution of interdisciplinary teams for reference and matrix support plays an important role, strengthening the bond, continuity and coordination of care^(24)^.

It is clear that facing underfunding and privatization requires in-depth and up-to-date knowledge on these themes^(16)^, with a view to subsidizing popular participation and the efforts of movements in defense of the SUS^(22-24)^. Therefore, this paper is part of this perspective.

With regard to the Health and Nursing area, the need to highlight the role and power of the private actors in the elaboration and conduction of the public policies stands out, despite the relative invisibility for broad social segments of their particularistic and market motivations, which implies rethinking strategies to guarantee the maintenance and expansion of social rights^(25)^.

The current context is probably the most difficult ever experienced by SUS. The ultraliberal platform expressed, for example, in Constitutional Amendment 95/2016, freezes social spending for 20 years^(26)^, in the reformulation of the Primary Care policy – reducing the priority of the Family Health Strategy –, in the proposals – always present – of privatizing the management of the public services, including Primary Health Care, and the dismantling of the Mental Health Policy, among others, hinders any possibility of SUS survival as a truly public, universal and comprehensive system. Those who advocate for SUS – the population including health workers and the academics – are more than ever responsible for the undeniable task of sustaining the advances of the Brazilian system against the neoliberal offensive; not as uncritical acceptance of the stage reached, but as the level from which an expanded movement must be organized, with the objective of overcoming the limits of SUS, in order to guarantee comprehensive care in fact as an inalienable human right.

In conducting this study, some limitations must be considered. The population data were not incorporated into the analysis of the dependent variables. In addition, subgroups of procedures and the predominant care profile in each regime; the regional heterogeneities and their implications on the public-private arrangement; the possible differences in size between public and private hospitals and their implications on performance have not been evaluated. Therefore, it is suggested that these variables are analyzed in further research studies.

However, despite the limitations, it is believed that this study is a pioneer in the analysis of the public-private arrangement in surgical hospitalizations through SUS with national reach in the decade under study. It is hoped that the diverse evidence produced may contribute to the advancement of the scientific production on this theme. It is also worth noting that most of the studies aimed at discussing health privatization concentrate on supplementary health. There are few studies devoted to the analysis of the private presence complementary to SUS^(16)^, as the proposed here.

In addition, the diverse evidence generated can contribute to deepening reflection and debate, since the international and national actors and policies aimed at restricting social rights and privatizing public policies, including health, are still poorly investigated in Brazil^(25)^.

## Conclusion

The fact, that during the analyzed period, the surgical hospitalizations in the SUS were carried out predominantly by private services, which presented a higher average value of hospitalization and absorbed most of the resources spent in this area, expresses how much, in this sphere, the public sector is presented as complementary, in opposition to the constitutional principles. At the same time, it was verified that the average stay and mortality rate were higher in the public sector, with a statistically significant difference between public and private for all the variables analyzed.

Therefore, it is hoped that this study may contribute to a critical reflection on the ongoing process of privatization and mercantilization of health in Brazil. The aim is to subsidize actions in order to avoid budgetary asphyxiation of the public sector in favor of the private sector, which feeds the capital accumulation cycle through the social policies and the transfer of the public fund, with significant implications for access and care.

## References

[1] Meara JG, Leather AJ, Hagander L, Alkire BC, Alonso N, Ameh E (2015). Global Surgery 2030: evidence and solutions for achieving health, welfare, and economic development.. Lancet. [Internet].

[2] Gyedu A, Stewart B, Gaskill C, Boakye G, AppiahDenkyira E, Donkor P (2018). Improving benchmarks for global surgery: nationwide enumeration of operations performed in Ghana.. Ann Surg. [Internet].

[3] Giovanella L, Mendoza-Ruiz A, Pilar ACA, Cantanhêde RM, Martins GB, Santos IS (2018). Universal health system and universal health coverage: assumptions and strategies.. Ciênc Saúde Coletiva. [Internet].

[4] Xu K, Soucat A, Kutzin J, Brindley C, Maele NV, Touré H (2018). Public spending on health: a closer look at global trends. [Internet].

[5] Massenburg BB, Saluja S, Jenny HE, Raykar NP, NGKamstra J, Guilloux AGA (2017). Assessing the Brazilian surgical system with six surgical indicators: a descriptive and modelling study.. BMJ Glob Health. [Internet].

[6] Covre ER, Melo WA, Tostes MFP, Fernandes CAM. (2019). Trend of hospitalizations and mortality from surgical causes in Brazil, 2008 to 2016.. Rev Col Bras Cir. [Internet].

[7] Ministério da Saúde (BR) (2010). Portaria nº 1.034, de 05 de maio de 2010. Dispõe sobre a participação complementar das instituições privadas com ou sem fins lucrativos de assistência à saúde no âmbito do Sistema Único de Saúde. Diário Oficial da União, Brasília, DF.

[8] Oliveira PR, Guerra MG, Oliveira A, Martins AL. (2019). Publicprivate relation in the Brazilian policy of tertiary care for cardiovascular conditions.. Rev Adm Pública. [Internet].

[9] Ministério da Saúde (BR) (2018). DATASUS. [Homepage].

[10] Viacava F, Oliveira RAD, Carvalho CC, Laguardia J, Bellido JG. (2018). SUS: supply, access to and use of health services over the last 30 years.. Ciênc Saúde Coletiva. [Internet].

[11] Noronha KVMS, Guedes GR, Turra CM, Andrade MV, Botega L, Nogueira D (2020). The COVID-19 pandemic in Brazil: analysis of supply and demand of hospital and ICU beds and mechanical ventilators under different scenarios.. Cad Saúde Pública. [Internet]..

[12] Braga FC, Barbosa PR, Santos IS., Giovanella L (2017). Políticas e Sistema de Saúde no Brasil. 2ª reimpressão.

[13] Confederação Nacional de Municípios (BR) (2018). Brasil perdeu 23.091 leitos hospitalares em dez anos. [Internet].

[14] Machado JP, Martins M, Leite IC. (2016). Public-private settlement and hospital mortality per sources of payment.. Rev Saúde Pública. [Internet].

[15] Silva JFM, Carvalho BG, Domingos CM. (2018). Health governance and the public-private relationship in small municipalities.. Ciênc Saúde Coletiva. [Internet].

[16] Bahia L, Scheffer M. (2018). The Unified Health System (SUS) and the private assistance sector: interpretations and facts.. Saúde Debate. [Internet].

[17] Bahia L. (2018). Thirty years of history in the Brazilian Unified National Health System (SUS): a necessary but insufficient transition.. Cad Saúde Pública. [Internet].

[18] Martins AL, Guerra M, Oliveira MS. (2019). Public-private relationship in the Brazilian policy of tertiary care for cardiovascular conditions.. Rev Gestão Saúde [Internet]..

[19] Conill EM., Campos GWS, Minayo MCS, Akerman M, Drumond M, Carvalho YM (2006). Tratado de saúde coletiva.

[20] Celuppi IC, Geremia DS, Ferreira J, Pereira AMM, Souza JB. (2019). 30 years of the SUS: public-private relationship and the impasses for the universal right to health.. Saúde Debate. [Internet].

[21] Cárdenas WIL, Adelyne MMP, Cristiani VM. (2017). Publicprivate relations in the Colombian health system from 1991 to 2015.. Cad Saúde Pública..

[22] Paim JS. (2018). Thirty years of the Unified Health System (SUS).. Ciênc Saúde Coletiva [Internet]..

[23] laurell AEC (2016). Competing health policies: insurance against universal public systems. Rev. LatinoAm. Enfermagem [Internet].

[24] Campos GWS. (2018). Future prospects for the SUS.. Ciênc Saúde Coletiva. [Internet].

[25] Almeida C. (2017). Public-private partnerships (PPPs) in the health sector: global processes and national dynamics.. Cad. Saúde Pública [Internet].

[26] Mariano CM. (2019). Emenda Constitucional 95/2016 e o teto dos gastos públicos: Brasil de volta ao Estado de exceção econômico e ao capitalismo do desastre.. Rev Investig Constit. [Internet].

